# Supplementary figures and images for: The transcriptional and translational landscape of HCoV-OC43 infection
Source: PLoS Pathog. 2025 Jan 27;21(1):e1012831. doi: 10.1371/journal.ppat.1012831 (PMC11771880; doi:10.1371/journal.ppat.1012831)

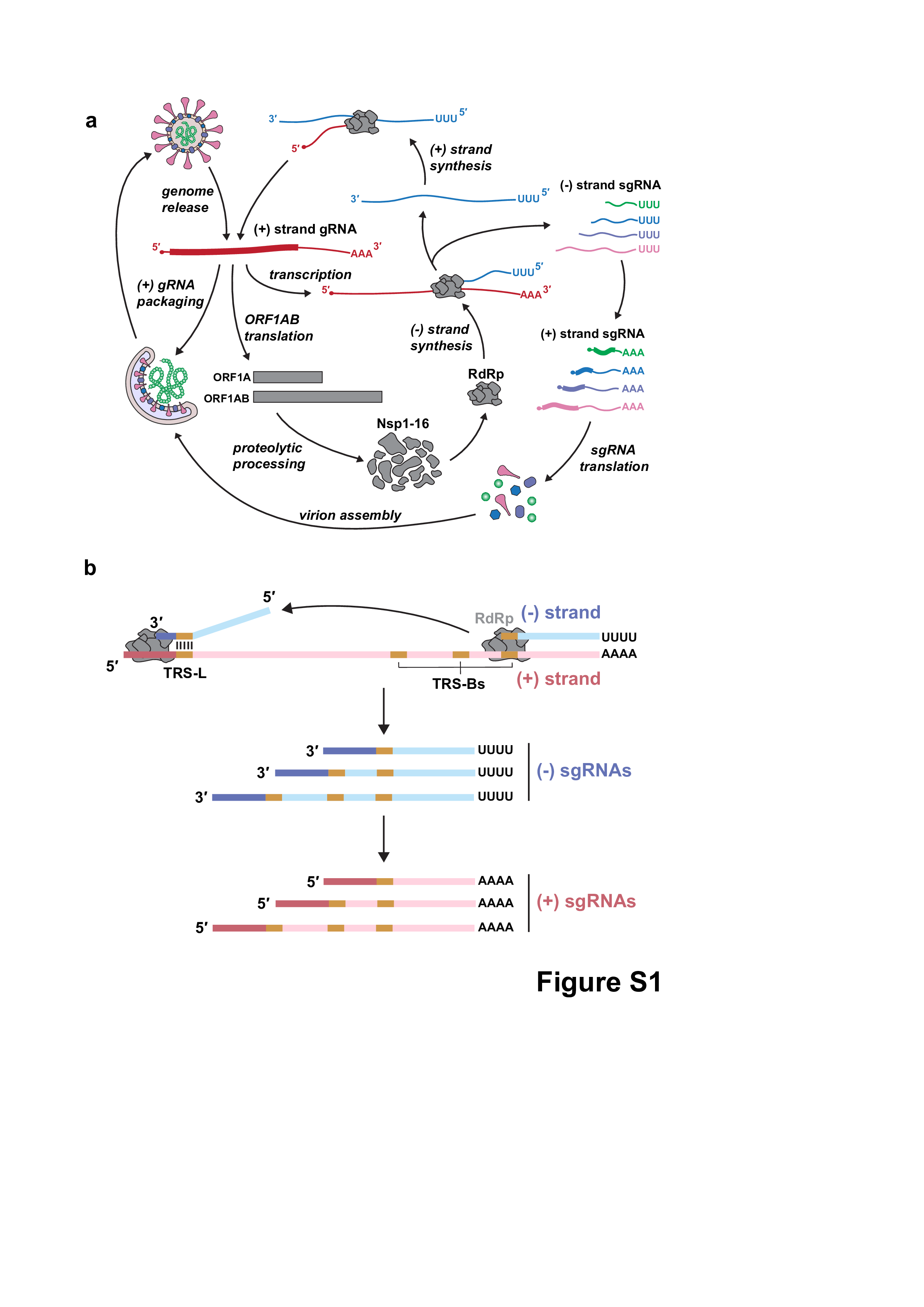

Supplement: S1 Fig — (a) The viral life cycle. See Introduction for details. (b) Schematic outline of discontinuous transcription. The viral genome incorporates TRS-B motifs upstream of each structural and accessory protein ORF. When RdRp transcribes a TRS-B, it sometimes “jumps” to the TRS-L at the 5ʹ end of the genome. Because the TRS-L and each TRS-B are similar in sequence, the nascent RNA reanneals to the template, and transcription resumes. The relative abundance of each (−) sgRNA is determined by how frequently RdRp jumps at each TRS-B. In the final step, the (−) sgRNAs serve as templates for synthesis of (+) sgRNAs which are subsequently used in translation. (TIFF) [file ppat.1012831.s001.tiff]

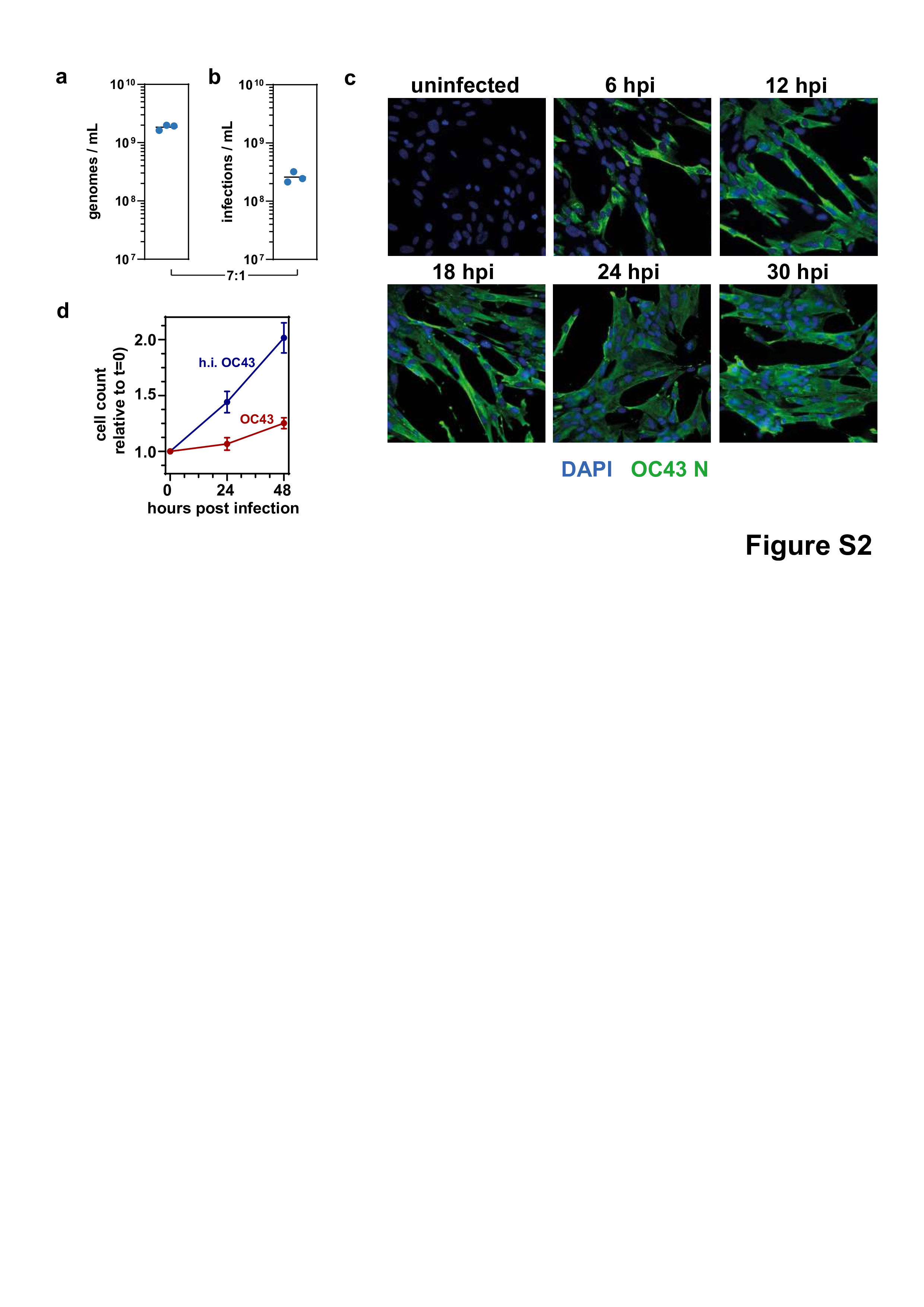

Supplement: S2 Fig — Related to Fig 1. (a and b) Preparation of the viral stock. To minimize experimental variability, we prepared a single batch of virus for the duration of the study. (a) Viral genomes were quantified using RT-qPCR against a region within ORF1A. (b) Infectious particles were quantified using limiting dilution and assessment of cytopathic effect (CPE) under the experimental conditions used in the study. The resulting particle:infection ratio was 7:1. (c) Immunofluorescence microscopy showing infected cells throughout the time course. Cells were stained with DAPI to visualize cell nuclei and decorated with an antibody against the viral N protein to identify infected cells. (d) Cell growth rates following infection with OC43 or mock-infection with heat-inactivated (h and i) virus. Error bars show the standard deviation around the mean (n = 3). (TIFF) [file ppat.1012831.s002.tiff]

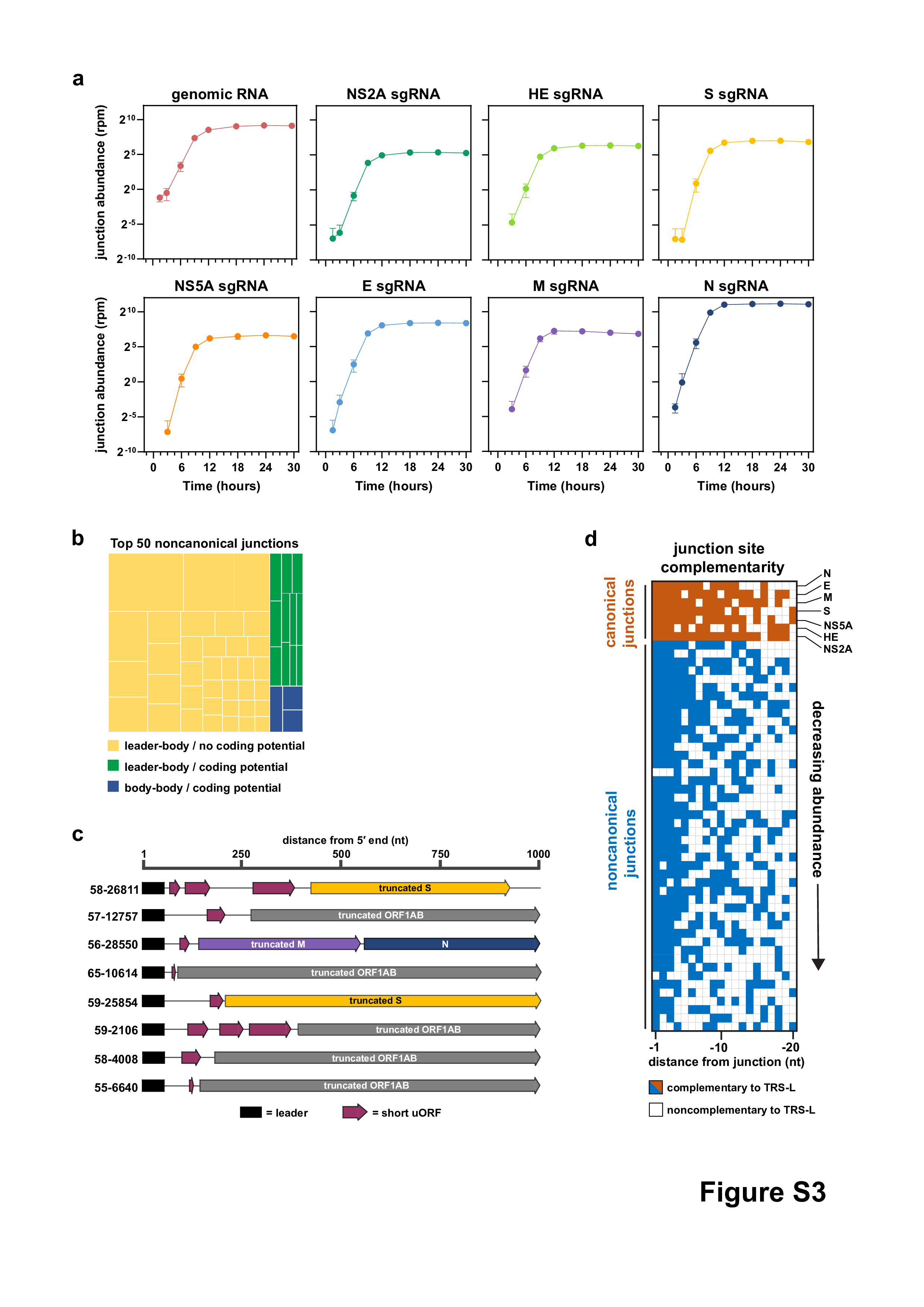

Supplement: S3 Fig — Related to Fig 2. (a) Plots showing the expression kinetics of genomic RNA and individual sgRNAs throughout the time course. Error bars show standard deviation of the mean (n = 4). (b) Tree diagram showing the structure of the 50 most abundant noncanonical sgRNAs. (c) Structure and protein coding potential of the eight most abundant noncanonical sgRNAs. In every case, the initial ORF is no longer than 16 amino acids. The numbers indicate the donor-acceptor coordinates. (d) Plot showing the hybridization potential between donor sites (i.e., TRS-Bs) and the TRS-L acceptor site. Transcripts are sorted by abundance. Complementary bases are displayed in orange (canonical junctions) or blue (noncanonical junctions), and noncomplementary bases are shown in white. (TIFF) [file ppat.1012831.s003.tiff]

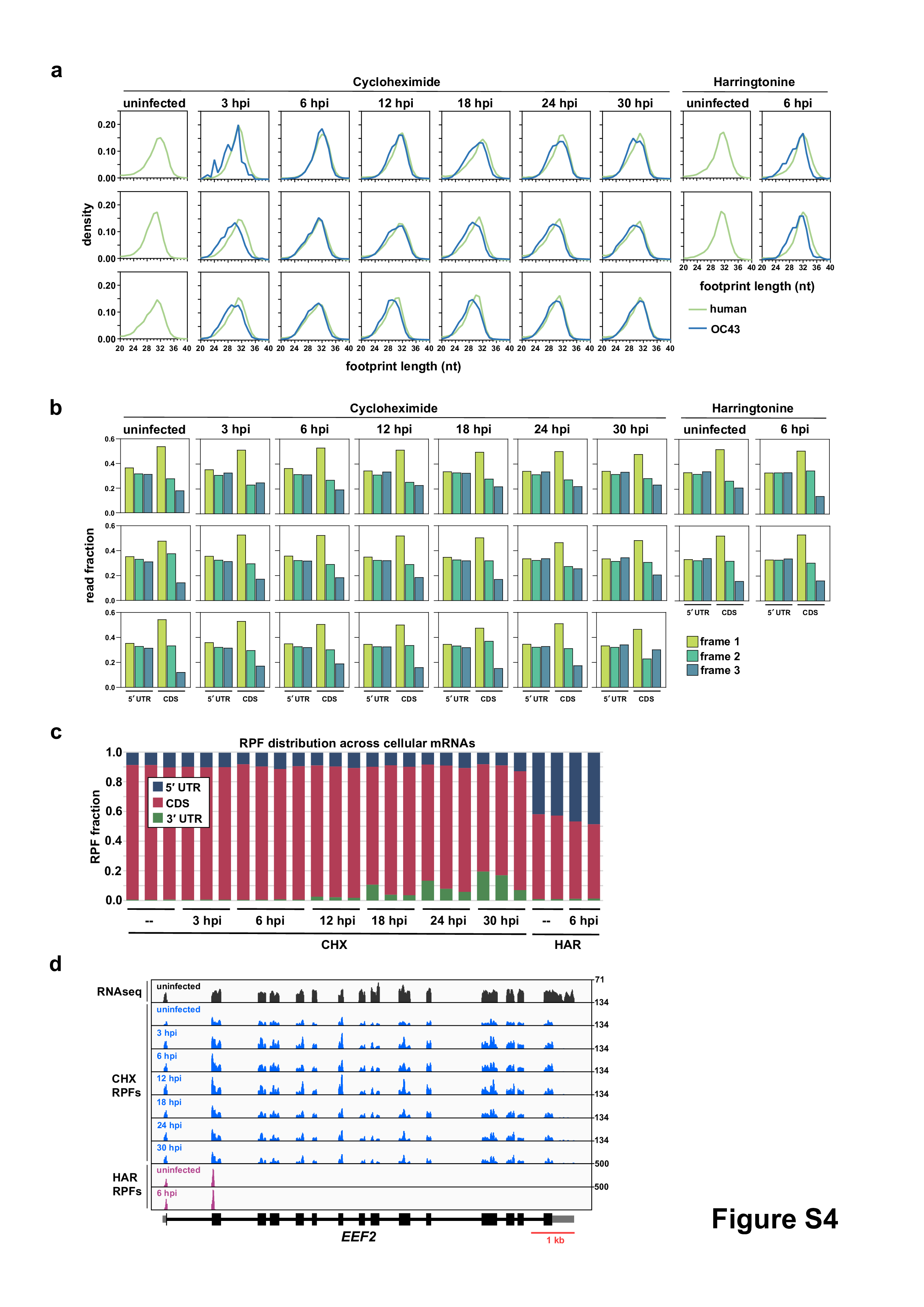

Supplement: S4 Fig — Related to Fig 3. (a) Length distribution of RPFs mapping to cellular (green) and viral (blue) CDS regions following gel-based selection for 26–34 nt fragments. (b) Proportion of cellular-mapping RPFs associated with each reading frame. (c) Distribution of Riboseq reads between 5ʹUTR, CDS, and 3ʹUTR regions. (d) Riboseq read densities across the EEF2 gene for a representative replicate. Total RNA (black), CHX RPFs (blue), HAR RPFs (pink). The numbers to the right of the plot show the scale in RPM for each track. The annotation for EEF2 is shown below. Grey rectangles show 5ʹ and 3ʹUTR regions, black rectangles show CDS, and black lines show introns. (TIFF) [file ppat.1012831.s004.tiff]

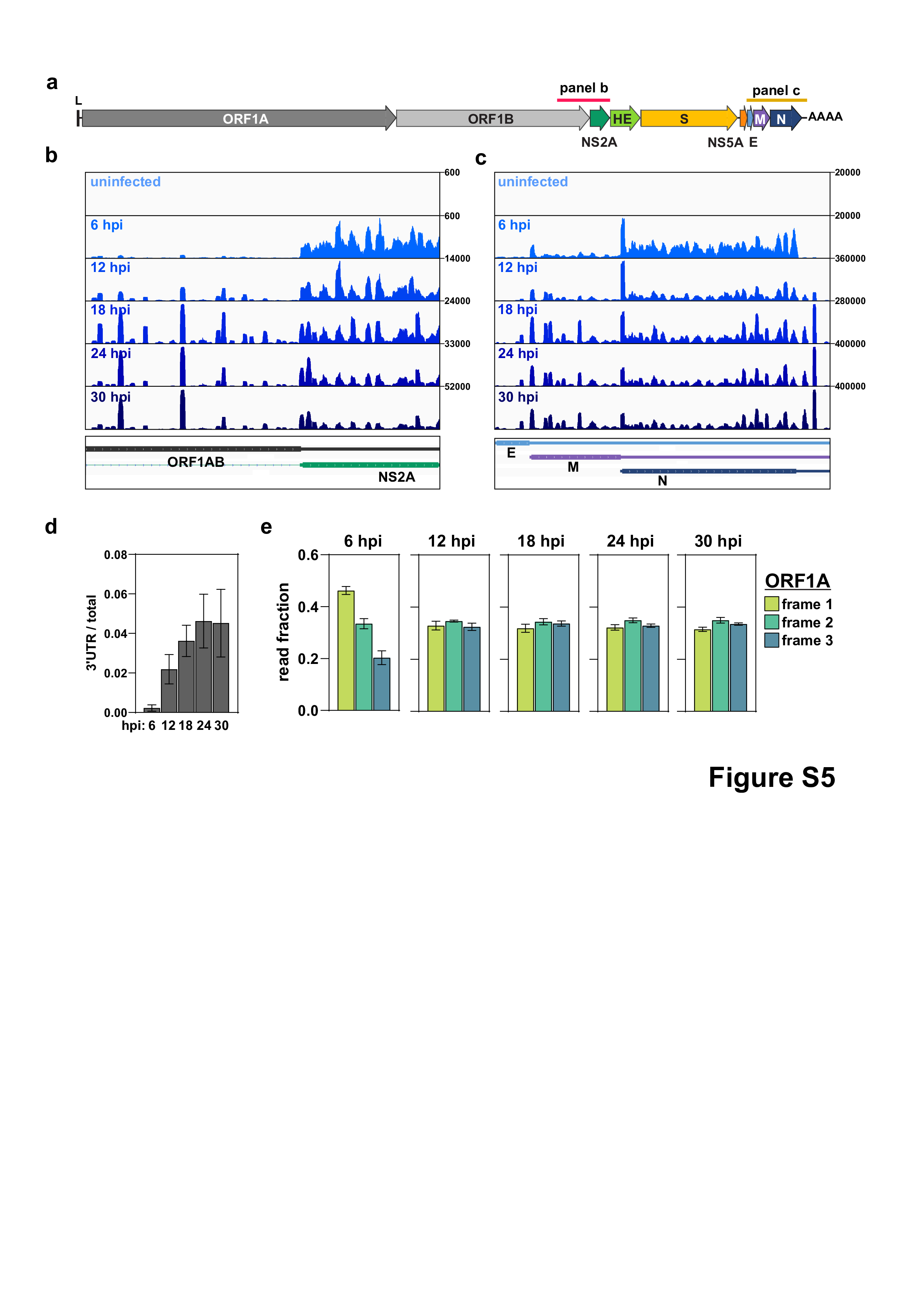

Supplement: S5 Fig — Related to Fig 3. (a) Schematic showing the structure of the full-length viral gRNA. The scale bars below the annotation highlight the regions displayed in panels (b) and (c). (b and c) Ribosome density across sections of the viral genome. (d) The relative proportion of viral-mapping RPFs which map to the 3ʹUTR. Error bars show standard deviation of the mean (n = 3). (e) Proportion of viral-mapping RPFs associated with each reading frame across ORF1A. Error bars show standard deviation of the mean (n = 3). (TIFF) [file ppat.1012831.s005.tiff]

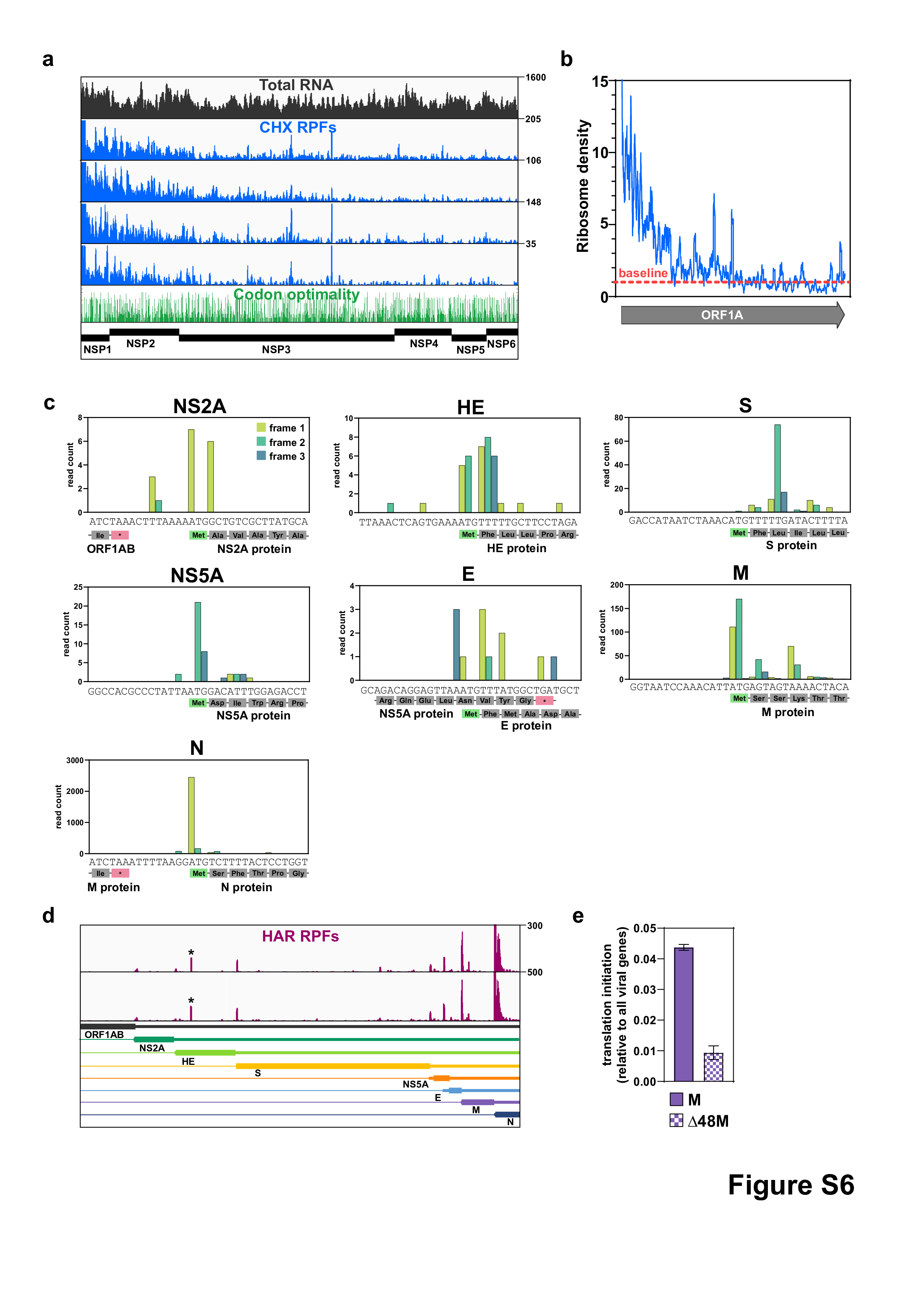

Supplement: S6 Fig — Related to Fig 3. (a) Ribosome density at 6 hpi across a section of ORF1A. Four independent replicates of CHX Riboseq are shown (blue). Total RNA determined by RNAseq is shown as a comparison (black). Codon optimality scores are shown below (green). (b) Ribosome density across ORF1A. The red baseline shows the average ribosome density across the latter half of ORF1A. (c) Distribution of HAR RPFs around the start codons of each canonical viral ORF. (d) Density of HAR RPFs at 6 hpi across the subgenomic region of the viral genome. Two independent replicates are shown. The asterisk marks a likely sequencing artifact. (e) Bar chart showing the relative proportion of RPFs associated with the primary start codon for M versus the downstream initiation site. Error bars show the standard deviation around the mean (n = 2). (TIFF) [file ppat.1012831.s006.tiff]

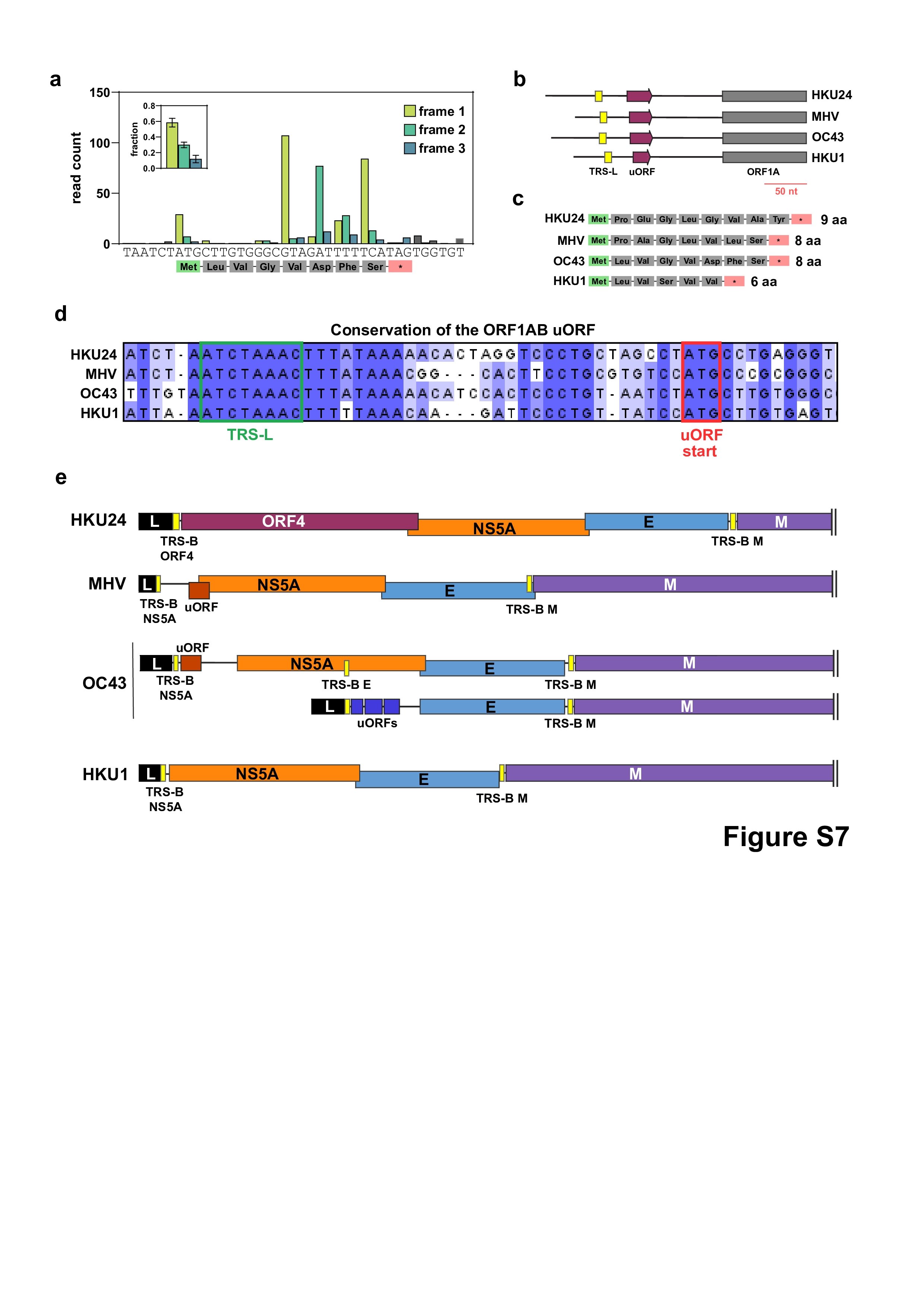

Supplement: S7 Fig — Related to Fig 3. (a) P-site mapping for CHX RPFs across the ORF1AB uORF. Below: the codon and amino acid sequence for the ORF1AB uORF. (b and d) Schematic showing the conserved architecture (b), peptide sequences (c), and sequence context (d) of the ORF1AB uORF across the betacoronavirus A lineage. (e) Genomic organization of the NS5A and E genes across various representatives of the betacoronavirus A lineage. The architectures for MHV [5] and OC43 were determined experimentally, and HKU24 and HKU1 were inferred based on the presence or absence or TRS-B motifs. (TIFF) [file ppat.1012831.s007.tiff]

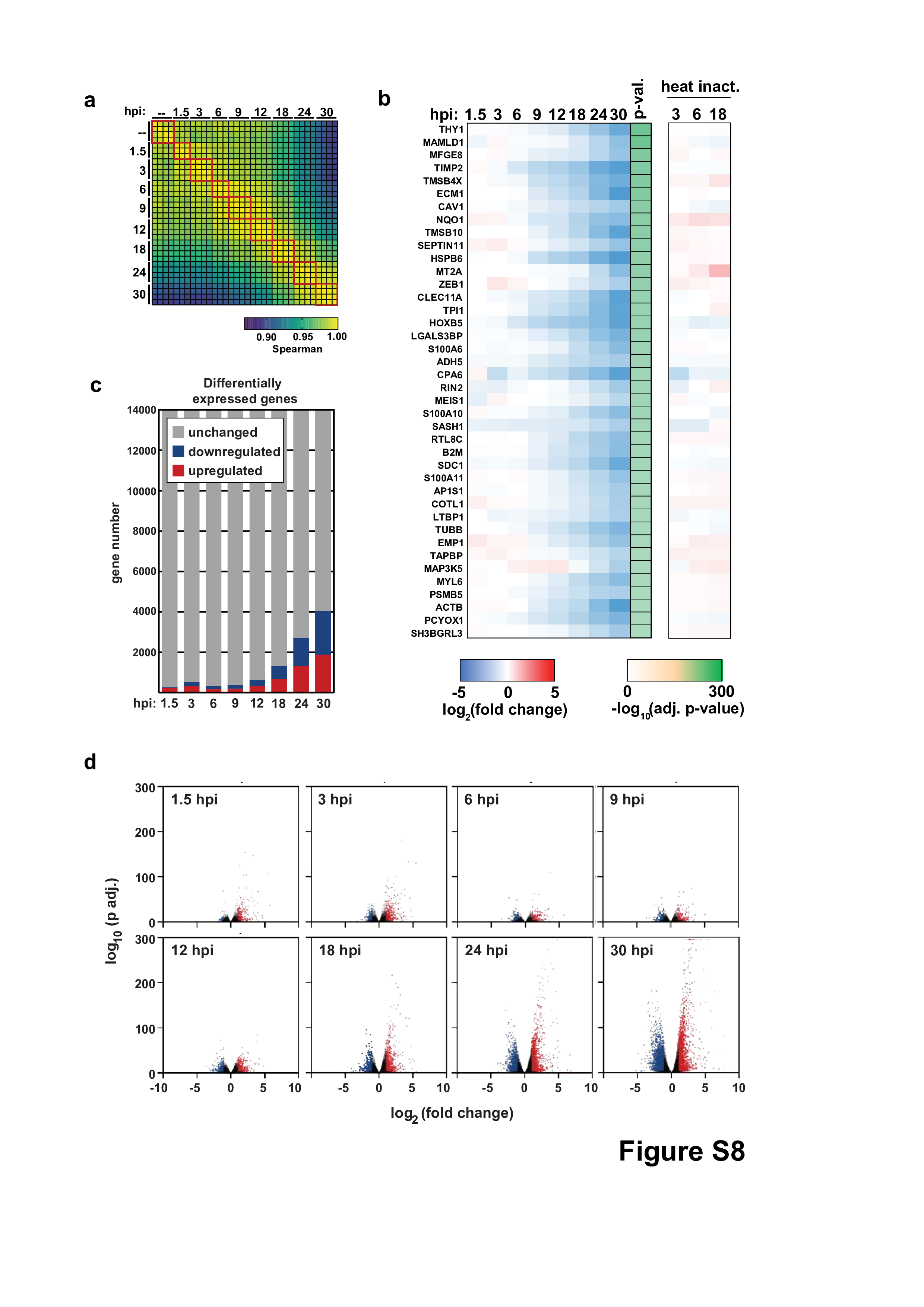

Supplement: S8 Fig — Related to Fig 4. (a) Matrix showing the pairwise Spearman correlation coefficients for human transcripts between different replicates and timepoints. The red squares outline each set of replicates. (b) Heatmap showing the changes in RNA abundance throughout the time course. The heatmap shows the most significantly decreased transcripts (by p-value). (c) Bar chart showing the number of differentially expressed genes (DEGs) at each timepoint. (d) Volcano plots showing statistically significantly upregulated (red) and downregulated (blue) genes at various timepoints after infection. (TIFF) [file ppat.1012831.s008.tiff]

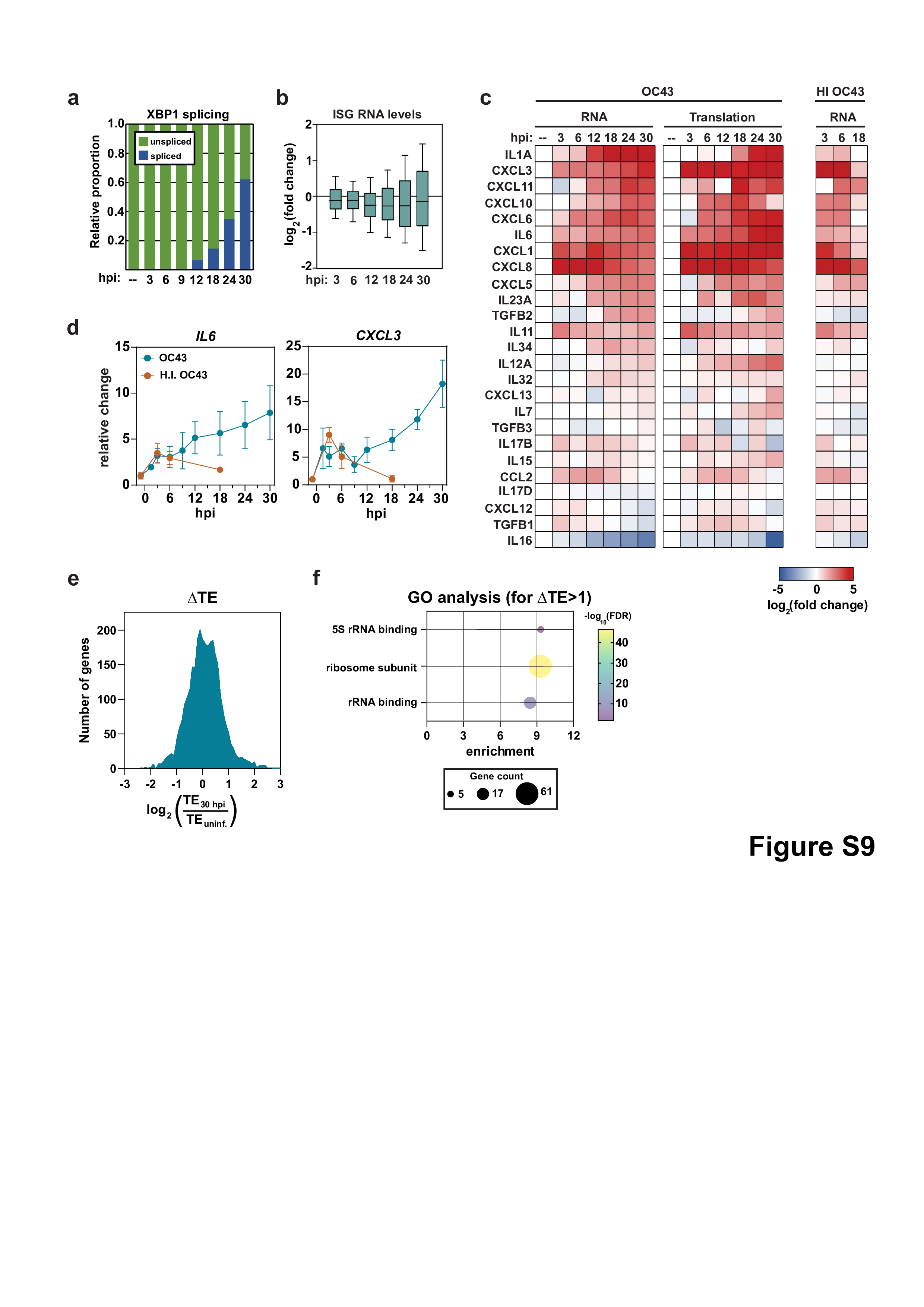

Supplement: S9 Fig — Related to Fig 4. (a) Bar graph showing the relative proportion of spliced and unspliced XBP1 throughout the time course. (b) Box-and-whisker plot showing the expression of interferon stimulated genes (ISGs) in response to infection. The box shows the 25th, median, and 75th percentile, while the whiskers show the 10th and 90th percentiles. (c) Transcriptional (left) and translational (center) induction of cytokines and chemokines in response to infection. The transcriptional response to mock infection with heat-inactivated (HI) virus is also shown (right). (d) Changes in mRNA abundance and translation during the infection time course for two cytokine genes. Error bars show the standard deviation around the mean (n = 3–4). (e) Histogram showing the change in translation efficiency (TE) following infection. The 3000 most-expressed genes are included in the analysis. (f) GO analysis for genes showing an increase in TE (log2≥1). (TIFF) [file ppat.1012831.s009.tiff]

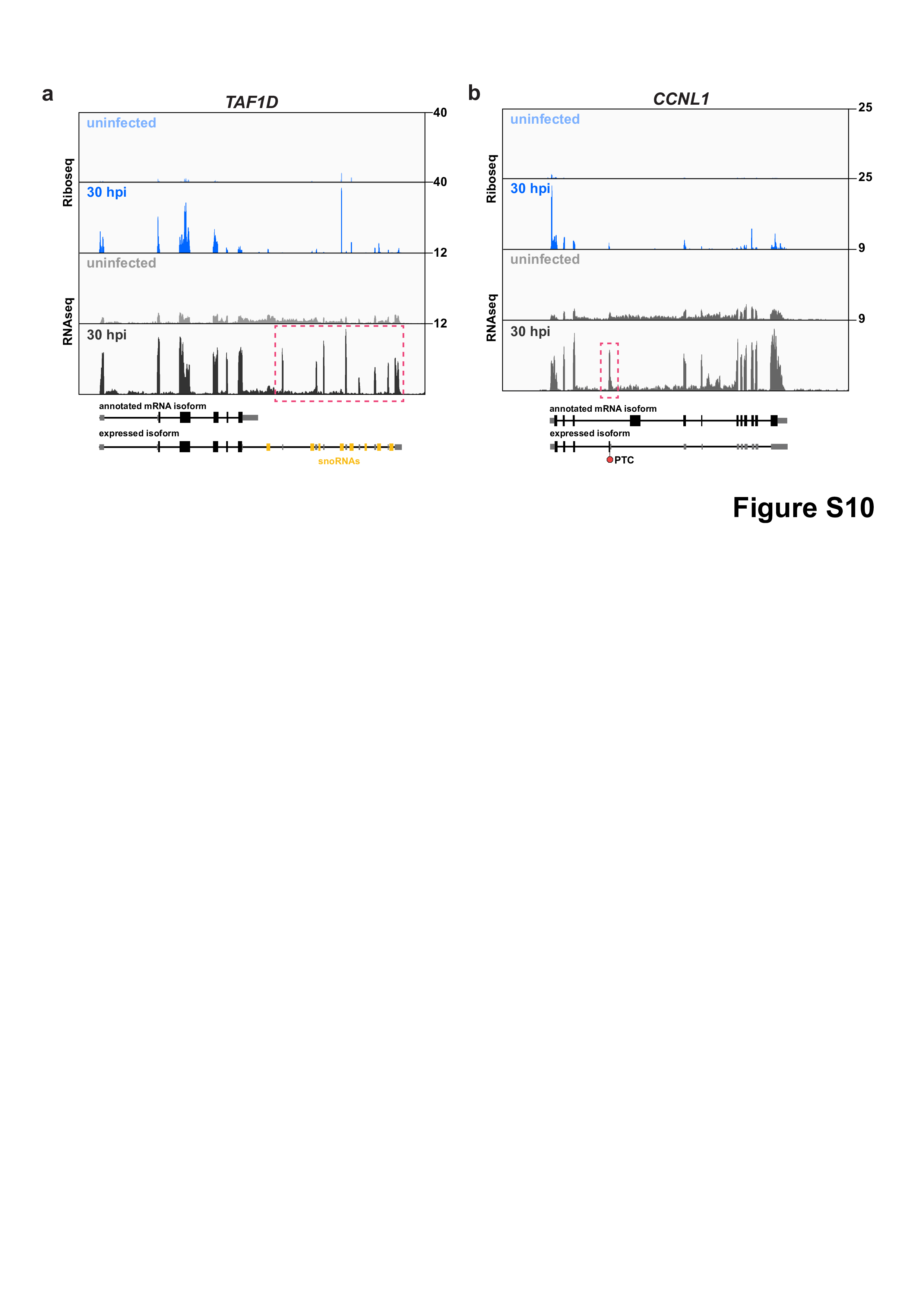

Supplement: S10 Fig — Related to Fig 5. (a and b) Ribosome density (blue) and RNA abundance (grey) across TAF1D (a) and CCNL1 (b). Numbers to the right indicate the scale in RPM. Note that TAF1D includes eight additional exons beyond the normal stop codon, while CCNL1 incorporates an unannotated exon that includes a PTC. Both features are highlighted with a dashed red box. (TIFF) [file ppat.1012831.s010.tiff]
