# Supplementary material for: The transcriptional and translational landscape of HCoV-OC43 infection
Source: PLoS Pathog. 2025 Jan 27;21(1):e1012831. doi: 10.1371/journal.ppat.1012831 (PMC11771880; doi:10.1371/journal.ppat.1012831)
Supplement: Attachment — Submitted filename: 143683_1_rebuttal_3188815_slyd9b.docx [file ppat.1012831.s017.docx]

**Manuscript number:** **RC-2024-02360**

**Corresponding author(s):** **Stefan Bresson**

*[Please use this template only if the submitted manuscript should be considered by the affiliate journal as a full revision in response to the points raised by the reviewers.*

*If you wish to submit a preliminary revision with a revision plan, please use our "*[*Revision Plan*](https://docs.google.com/document/u/0/d/1HHp62Tfeb44nd3Gu_3ChB0b8gmMdEog7GjZcZFwHSiQ/edit)*" template.* ***It is important to use the appropriate template to clearly inform the editors of your intentions.****]*

## 1. General Statements [optional]

## We are submitting a fully revised version of the manuscript in response to reviewer comments and hope that the revised version of the manuscript is appropriate for publication in eLife. In the point-by-point description below, reviewer comments are in black, and our response is in blue.

## Point-by-point description of the revisions

*This section is mandatory.* *Please insert a point-by-point reply describing the revisions that were already carried out and included in the transferred manuscript.*

Reviewer #1 (Evidence, reproducibility and clarity (Required)):

In this manuscript the authors rather comprehensively analyzed the transcriptional and translational landscape of HCoV-OC43 infection over a 30 hour time course using RNAseq and Riboseq (ribosome profiling using both CHX and harringtonine) analyses. Due to apparent technical issues with viral RNA packaging, the Riboseq analysis was focused on the 6 hpi time point. Interesting findings included slow translation rates in the last 3/4ths of the large ORF1A on gRNA, a frameshift efficiency of ~56% from ORF1A to ORF1B - consistent with other coronaviruses studied to date, and a variety of noncanonical translation across the OC43 genome, several of which appear to be unique to this specific coronavirus. Changes in cellular gene expression during infection (performed by RNAseq analyses) demonstrated ~30% of the host transcriptome either up or down regulated. The top GO terms associated with this dysregulated all pointed to ER stress. Interestingly, interferon genes were not induced and several genes (conventional mRNAs and lncRNAs with short ORFs) were noted to escape NMD surveillance.
Overall, the experimental design is straightforward, the conclusions are well-supported by the data, and the manuscript reports foundational information for understanding molecular aspects of an OC43 infection. I feel that the manuscript is thus a significant contribution to the field. I only have a couple of minor suggestions to polish the manuscript:

Minor suggestions:

1. Fig. 4e; Fig. 5c and f: I would recommend including a statistical analysis for these data to determine significance.

**We now include a statistical analysis for these figure panels.**

2. I would recommend introducing MRC-5 cells as lung fibroblasts at the beginning of the results section since all readers may not recognize these cells without a description.
**The referee makes a good point. We have modified the text to indicate this.**

Reviewer #1 (Significance (Required)):

The manuscript reports foundational information for understanding molecular aspects of an OC43 infections in MRC-5 cells


Reviewer #2 (Evidence, reproducibility and clarity (Required)):

In this manuscript, the authors analysed the transcriptional and translation landscapes of human lung fibroblasts during infection by the coronavirus OC43. They found previously unannotated ORFs. They could also monitor the activation of the genes involved in stress response. In contrast, the genes involved in anti-viral responses remained silent. In addition, the authors concluded that the Non-sense-Mediated Decay (NMD) pathway was somehow shut down thereby leading to the accumulation of noncoding transcripts. Overall, this study brings an impressive amount of interesting data on the impact of OC43 infection on the cell metabolism. The manuscript is very well written and illustrated by informative and well-designed figures. The data are generally properly interpreted. Although the results are only descriptive, the brought conclusions are clear and of general interest for the virus community. However, a few specific points should be improved for full acceptance of the manuscript. For instance, the effect of viral infection on NMD is very interesting and should be further documented.


Specific points

- No statistics in fig 3l. To rule out that the presence of the uORF is indeed critical for translation efficiency, the mutations of the uAUG should be tested in NS5A and E constructs. The mutations are expected to rescue the translation efficiency.

**We now indicate statistical significance within the figure panel.**

**We repeated the luciferase reporter experiment to include versions of the NS5A and E reporters in which the uORF(s) are disrupted. Surprisingly, the ΔuORF variants were not translated any more efficiently than their wild type counterparts. This may suggest that the uORFs are not primarily regulating translation of the downstream ORF, but future work will be needed to confirm this.**

- The reads for the uORF that is located in the leader sequence (shown in Fig 7a) are not equally detected. For instance, it is puzzling that aa #2, 3 and 4 are not seen and that the frame is lost for aa #6 and 7. The authors should at least comment this point.

**The referee makes a good point. We have modified the text to indicate that the evidence for translation of the ORF1AB uORF is not definitive. We have provided a similar caveat for E uORF#1, which is also not so clear-cut.**

- The presented data suggest that NMD substrates are stabilized upon infection, suggesting that the NMD machinery becomes inactivated by infection. This is an interesting conclusion that should be further investigated by measuring the expression, the presence and the abundance of NMD machinery components during infection. In addition, this point should be discussed in more details in the discussion section.

**The increased expression of NMD substrates does not appear to be caused by a decrease in the expression levels of genes encoding various NMD factors. If anything, the opposite is true for UPF1, which shows a significant increase in expression at the RNA and translation level (see below). Source data can be found in Tables S2 and S5.**

**This does not exclude the possibility that protein expression is directly regulated, independent of changes in transcription and translation. We recently used pulse-chase SILAC labeling to follow global protein synthesis and turnover rates during the course of OC43 infection. This set of experiments is currently being prepared for separate publication, as the current manuscript already seems overloaded with datasets. However, to address the reviewer’s question, we mined our proteomic datasets to determine whether major NMD factors show any significant decrease upon infection. Below, we show the protein abundance of five different NMD factors at 24 hours following infection with either active OC43 virus or a heat-inactivated negative control. We observed a modest decrease in the expression of Upf1 and Smg5, though it seems unlikely that this is sufficient to explain the apparent suppression of NMD during infection.**

**A possible explanation may come from a recent publication (PubMed ID: 39360627). The authors found that the SARS-CoV-2 nucleocapsid protein binds UPF2 and prevents it from activating UPF1. We speculate that the OC43 nucleocapsid protein may work in a similar manner. We now discuss this paper and its implications for our work in the Discussion section.**

Minor points:
- p8, the main text mentions Fig4f, but the figure 4 does not have a 4f panel.
**Thanks, this has now been corrected.**

Reviewer #2 (Significance (Required)):

The manuscript brings novels insights in the cellular metabolism during HCoV-OC43 infection. In addition, the techniques used allow a precise assessment of viral translation from both genomic and subgenomic RNAs. The presented data are original and bring novel insights on OC43 infection in human fibroblasts. The results are definitely of general interest for the broad RNA biology community and virologists.

Reviewer #3 (Evidence, reproducibility and clarity (Required)):

In this manuscript, Bresson et al perform RNA-seq and ribosome profiling to characterize transcription and translation, respectively, in human lung fibroblasts infected with betacoronavirus OC43, a relative of SARS-CoV-2 that causes common cold symptoms. The authors offer a comprehensive analysis of OC43 transcription and translation, including previously annotated as well as new sgRNAs and open reading frames. Finally, the authors analyze the host response to infection and find that ER stress is triggered, nonsense mediated decay is inhibited, and innate antiviral responses-but not chemokines and cytokines-are silenced.

Overall, the data is of high quality and can likely be used as a resource for research involving the regulation of transcription and translation during coronavirus infection. However, I struggled with the number, density and interpretation of data panels in both main and supplementary figures, as well as the flow of the text. The manuscript is dense in data but sparse in validated conclusions, limiting its significance. Instead of focusing on one or two interesting observations, the authors attempt to probe an unusually wide range of events related to infection: canonical and noncanonical sgRNA transcription, its molecular determinants and expression over time; 5'UTR organization of individual sgRNAs and their relative translation efficiency; canonical and novel translation start sites on viral RNAs; read size and distribution of elongating ribosomes on viral RNA, including variable density on coding regions and increased occupancy on 3'UTR; periodicity of ribosomes translating host and viral RNAs; host transcriptional and translational response to infection, including ER stress, chemokine and cytokine responses (including potential bystander effects), and NMD; alternative splicing and stability of host transcripts during infection.

The text and data visualizations could benefit from some simplification, consolidation, and refocusing, which would hopefully make the manuscript more reader friendly. Specific comments and concerns are listed below.

Major comments:

- Global translation levels: other coronavirus inhibit global translation initiation in a manner dependent on both viral Nsp1 and host cell type. The role of OC43 Nsp1 should at least be discussed and compared to other coronaviruses. The authors should also measure global translation rates during infection using polysome profiles and/or metabolic labeling, to determine whether OC43 infection inhibits translation in MRC5 cells. This is critical, for example, for interpretation of the data in Fig. 4d-e, because overall low translation rates could explain the lag between the transcriptional and translational induction of specific host transcripts.

**We have now added a paragraph on Nsp1 to the discussion section. A recent publication from the Steckelberg lab (PubMed ID: 37660941) found that OC43 Nsp1 suppresses translation through a similar mechanism to SARS-CoV-2 Nsp1. Curiously, however, reporter RNAs carrying the 5’ leader sequence of the OC43 genome are translationally repressed in the presence of Nsp1 (in contrast to the SARS-CoV2 Nsp1 and leader sequence pair). This may explain the relatively low translation efficiency of viral transcripts (see the revised Fig. 3e).**

**We measured global protein synthesis using pulse-chase SILAC labeling, and found that protein synthesis is decreased ~50% between 12-24 hours post infection, suggesting translation is suppressed, but not completely abrogated. This is consistent with data from the paper above showing that OC43 Nsp1 imposes a relatively weaker translational suppression compared to SARS-CoV-2 Nsp1. However, we planned to include this in a separate publication, as the existing manuscript already appears overloaded with data.**

- Synchronization of infection: despite high MOI, images seem to show that, at 6 hpi, only a fraction of cells are positive for OC43 N protein (S2c), suggesting that infection may not be synchronized. The authors should quantify N-positive cells at each timepoint and address this lack of synchrony as a potential confounding factor. This is critical, because RNA-seq and ribo-seq average millions of cells, and if these include a significant fraction of uninfected cells, it will be impossible to determine whether any changes occur in infected or bystander cells.

**The viral titers were rigorously measured and highly reproducible, so we are confident that the infection parameters are correct.** **The seemingly inconsistent IF results at 6 hpi are almost certainly due to cell-to-cell heterogeneity in the speed with which cells reach a threshold level of infection. There are two possible reasons for this:**

1) **With an MOI of 7, we can expect 99.9% of cells to be infected with at least one infectious particle, but some cells will receive considerably more or less than 7. Cells which receive a higher dose of virus will inevitably appear ‘more infected’.**
2) **Some cells are inherently more or less permissive to infection, resulting in highly variable viral loads from one cell to another (see, for example,** [**https://elifesciences.org/articles/74153**](https://elifesciences.org/articles/74153)**).**

**We have now acknowledged this caveat within the revised manuscript.**

- Irregularity of ribosome protected reads: the observations that ribosome protected reads are (a) larger than usual, (b) lose periodicity on viral RNAs beyond 6 hpi and (c) are unevenly distributed on ORF1 (e.g. S5b, S6a-b) are worrying, as they likely represent technical rather than biological issues. At a minimum, the authors should provide a more detailed analysis of the relationship between footprint size and periodicity. A size window of 26-34 nt is unusually wide, and the extreme sizes may be products of RNA protected by e.g. replication or packaging. How does the periodicity analysis, particularly of viral sequences and particularly at later timepoints, change if the authors filter for reads e.g. 28-29 or 30-31 nt?

**Below, we provide a more detailed analysis of footprint size and periodicity for the 6, 12, and 24 hour timepoints. Footprints mapping to cellular mRNAs maintain periodicity throughout the timecourse, but footprints mapping to the virus lose periodicity after the 6 hour timepoint. This is true regardless of the specific footprint size.**

**The balance of evidence favors a biological explanation rather than technical issues, because the loss of periodicity is (1) specific to viral transcripts, and (2), only manifests at timepoints after 6 hours. Moreover, these patterns were highly reproducible across all three replicates.**

**As we note in the text, we think the loss of periodicity across the viral genome can be explained by genome packaging. We note that a similar explanation was put forward to explain a loss of periodicity across the SARS-CoV-2 genome at later timepoints (Finkel et al., 2020).**

**Finally, we would note that a size window of 26-34 nt is not unusually wide. For comparison, the SARS-CoV-2 ribosome profiling by Finkel et al. uses a similar size range (26-33 nt).**

- Furthermore, the uneven distribution of reads on ORF1a is reminiscent of the so-called 5' translation "ramp", which was attributed to either slower elongation or biases introduced by CHX. To reach any meaningful conclusion, a control without CHX would be useful.

**The “5’ translation ramp” was first reported in budding yeast, and refers to the observation that the first ~200 codons frequently show elevated ribosome density. However, this phenomenon has not been observed in mammalian cells, and in any case is independent of cycloheximide (see “Humans and other commonly used model organisms are resistant to cycloheximide-mediated biases in ribosome profiling experiments” by Sharma et al., 2021).**

**We also note that the uneven ribosome density we see for ORF1A is specific to that gene, and is not observed for any other viral ORF (see e.g. S5b-c), or cellular mRNAs. These results argue against a general technical bias caused by methodology.**

**Moreover, a similar decreasing gradient was reported for the 5’ end of ORF1A in MHV (PubMed ID: 26919232, and see below), a close relative of OC43. We now discuss this point in the revised version of the manuscript.**

The observation that infection is associated with increased RPF occupancy on 3'UTR of cellular transcripts, likely reflecting increased translational readthrough of stop codons, is also at odds with the authors' speculation that the observed 5' ramp is the result of premature termination.

**We considered the possibility that RPF occupancy in 3’UTRs reflects translational readthrough, but the available evidence argues against this. If ribosomes read through the natural stop codon at an elevated rate, they will eventually terminate at an alternative stop codon within the 3′UTR, leading to a gradual decrease in ribosome density throughout the 3′UTR. However, we observed elevated read density across the entirety of the 3′UTR. To the right, we show ribosome footprints across an individual transcript, but this example is typical.**

**These results suggest some other mechanism is involved. We also considered the possibility that ribosomes might initiate within the 3’UTR itself. This can happen if the 3′UTR is separated from the rest of the transcript (e.g. by endonucleolytic cleavage), allowing initiating ribosomes to bypass the normal open reading frame (for example,** [**https://pubmed.ncbi.nlm.nih.gov/33556964/**](https://pubmed.ncbi.nlm.nih.gov/33556964/)**). However, we were unable to demonstrate this directly. Ultimately, we decided not to pursue this further since it was tangential to the central claims in the paper. However, the apparent 3’UTR translation is an interesting phenotype which we do plan to investigate in the future.**

- Read counts/density: for some of their ribo-seq analyses, the authors show sparse read counts (e.g. S4d, S6c, 3j). Can meaningful conclusions be drawn based on just a handful of reads?

**The numbers to the top-right of each track reflect the scale, and not the total number of reads. For example, track 2 of S4d includes 100,000 reads, even though the scale is set to 153.**

Also, axis labels are missing from multiple panels, including 3c,d,f,h S5b,c, S6a,d).

**We have now added read count scales for each track.**

- For the reporter assays shown in Fig. 3k-l, the results should be normalized to intracellular reporter RNA levels, measured by qPCR.

**The referee makes a good point. We have now quantified the RNA abundance of each reporter RNA, and we present this alongside the luciferase activity in Figure 3L.**

Also, others have found that stability and translation efficiency of coronavirus UTRs varies depending on whether DNA or RNA is transfected (e.g. PMID 35849342, 38335237). Furthermore, it was shown that translation initiation from CoV2 gRNA and sgRNA 5'UTRs is different in infected and uninfected cells (34433827), further calling into question the interpretability of the results in Fig. 3.

**This is a good point and we now acknowledge these caveats in the revised version of the manuscript.**

- ER stress: the authors show that a subset of ER stress response genes is induced at the transcriptional and translational levels during infection. This induction could be triggered by the virus, to meet the biosynthetic needs of folding/processing viral proteins in the ER lumen. To confirm that such changes in gene expression result in ER stress, the authors should show additional evidence e.g. Xbp1 splicing, activation of IRE1 or PERK, phosphorylation of eIF2a or other well established hallmarks.

**We have now quantified XBP1 splicing throughout the infection time course. As expected, the results indicate that an increasing proportion of XBP1 is fully spliced at later timepoints, consistent with the onset of ER stress. These results are now included in the revised manuscript as Fig. S9a.**

**Interestingly, XBP1 is also one of the few examples of genes which show an increase in translation efficiency over the course of infection (see below).**

- NMD: this analysis is underwhelming and correlative, at best. The authors analyze 3 known NMD substrates (Fig. 5a-c, S10), plus several lncRNA, and conclude that NMD is inhibited. Additional work is required to (a) identify additional potential NMD substrates based on previously published work and test their expression in the data and (b) confirm that changes in expression are directly associated with NMD and not transcription, alternative splicing, or other modes of RNA degradation.

**The available evidence argues against the possibility that the changes in expression are due to changes in transcription or alternative splicing. We do not see any increase in the expression of unspliced precursor, as would be expected if the changes were driven by increased transcription. There is also no evidence for any change in splicing. The same fully spliced isoform is detectable both before and after infection; only the relative abundance changes. Taken together, this evidence is consistent with a reduction in decay rate. We cannot conclude with complete certainty that NMD is the decay pathway in question, but this is supported by two features: (1) these transcripts incorporate premature termination codons, and at least the SNHG transcripts are *bona fide* NMD targets, and 2) NMD has been shown to be suppressed by two other coronaviruses, including the closely related MHV. We therefore consider it justified to propose that the increased expression of NMD targets we observe is attributable to a suppression of NMD.**

**However, in response to the reviewer’s points, we highlight some potential caveats to our conclusions in the revised version of the manuscript.**

The authors also speculate that the increased ribosome occupancy on 3'UTRs, as detected during infection, should elicit an NMD response. However, this scenario is more likely to trigger nonstop mRNA decay (NSD).

**Nonstop mRNA decay is induced by translation of the poly(A) tail, whereas standard nonsense mediated decay can be triggered by regular 3’UTR translation (see e.g. PubMed ID: 21029861).**

- Translation efficiency (TE) analysis: studies in related viruses have found that some host transcripts are translationally silenced while others are induced. Instead of separately probing RNA and translation levels (e.g. Fig. 4d), the authors could attempt to analyze TE values, calculated by dividing ribo-seq and RNA-seq measurements for individual transcripts. Does translation efficiency of host transcripts change during infection? Are specific subsets of host transcripts repressed or induced at the level of translation?

**We now include two additional figure panels in Figure S9. Fig. S9e shows the changes in translation efficiency (TE) genome-wide, while S9f shows a GO analysis of the significantly-altered genes. We have also included an additional supplemental table to allow the reader to explore changes in TE across the genome.**

**We have also added details on translation efficiency to the heatmap in Fig. 4d. The additional column shows the change in TE between the uninfected condition and 30 hpi. Very few genes show a significant increase in TE. The most prominent exceptions are XBP1 and the uORF-containing gene PPP1R15A. Finally, we include a comparison between the translation efficiency of viral and cellular mRNAs at the 6 hour timepoint (Fig. 3e). The viral RNAs are translated relatively poorly compared to most cellular transcripts.**

How are ribosomes distributed on uORF-regulated transcripts e.g. ATF4, ATF5 and PPP1R15A?

**Below, we show ribosome density plots across the requested transcripts. In uninfected cells, nearly all translation is concentrated over the 5’UTR, as expected for genes regulated by uORF translation. By 30 hpi, the main ORF is translated at a significantly higher rate for ATF4 and PPP1R15A.**

**Grey = UTRs, Thick black = CDS, thin black = intron**

- The authors should expand the discussion to include a more detailed analysis comparing and contrasting the results of their ribo-seq analysis with those reported for other coronaviruses e.g. MHV (PMID 26919232) and SARS-CoV-2 (32906143, 35604092, 34433827).
**We now compare our results to these references throughout the manuscript.**

Figures:
- Many panels could benefit from better titles. This is particularly important because visualizations of RNA- and ribo-seq data are often similar (see e.g. 1e, 2a-c,g).

**We have now labeled these figure panels as either ‘RNAseq’ or ‘Riboseq’.**

- Fig. 1b: a similar plot for ribo-seq would be helpful.

**We initially made a similar plot for the riboseq data but we decided to omit it from the manuscript. The problem is that from ~12 hpi onward most riboseq reads map to the virus, but, as we explain in the text, these reads are unlikely to be *bona fide* ribosome footprints. We therefore felt that including this plot could be misleading.**

- Fig. 2b-c: these panels offer different visualizations of the same data. Consider moving one to the supplement.

**Fig. 2b and 2c show different things. Fig. 2b shows changes in sgRNA abundance, while Fig. 2c shows changes in the relative proportion of different viral RNAs.**

- Fig. 3a-b: were polysome profiles generated from uninfected MRC5 cells?

**Yes, we now clarify this point in the figure legend to 3a-b.**

- Fig. 3c-d: why is c, which is an inset of d, shown first?

**Fig. 3c is shown first because it is described first in the text.**

Also, why is the harringtonine trace not included in 3d? Finally, it may be helpful to include another inset for the leader region (similar to 1e). I couldn't find any panel that shows Harr RPF on ORF1 translation start site.

**We have updated Fig. 3d to include the harringtonine results, and added a separate inset showing the leader region.**

- Fig. 3e: how does this compare to translation efficiency of host transcripts?

**We have updated Fig. 3e to include the 2000 most-expressed cellular mRNAs. Translation of viral mRNAs is considerably less efficient than cellular mRNAs at 6 hours post infection. This could reflect inherently weak translation, or the possibility that a significant proportion of the viral RNA is inaccessible to the translation machinery (e.g. because it is involved in transcription or packaging).**

- Fig. 3f-g (and S6e): if ribosomes initiate at aa 48 of M sgRNA at a ratio of ~1:5, the density of CHX RPF on the canonical 5' terminus of M CDS should be lower. Is this the case?

**We did not observe reduced levels of CHX RPFs at the 5’ end (see below). This is likely explained by the general unevenness of Riboseq data, and that the expected difference of ~20% is relatively modest.**

Minor comments:

- Abstract: "we observed accumulation and increased translation of noncoding transcripts". If a transcript is translated, it must be coding. A better phrasing would be transcripts "annotated as long noncoding RNA (lncRNA)".

**We respectfully disagree with the reviewer that a translated RNA is, by definition, coding. Many well-known noncoding transcripts are translated to varying degrees, but are still referred to as ‘noncoding’ because they do not encode functional polypeptides. For example, numerous snoRNA host genes are targeted by NMD, a process which requires translation, yet these transcripts are still called ‘noncoding’ (see e.g. https://pubmed.ncbi.nlm.nih.gov/37783604/).**

- Introduction: "Each virus has a distinct complement of accessory, and sometimes structural, genes". All coronaviruses have structural genes that encode for virion components, as well as some combination of accessory genes and gene products.

**We understand that all coronaviruses encode structural proteins. The point being made was that complement of structural proteins can vary between different human coronavirus species. We have modified the text to make this point clearer.**

- Introduction: "This tug of war battle plays a key role in SARS-CoV-2 infection and pathogenesis, but remains poorly characterized for other coronavirus species". Please cite relevant literature for SARS-CoV-2, MERS and MHV.

**We have now added several citations covering all three coronaviruses.**

- Introduction: "We identified numerous noncanonical transcripts". Please provide a number.

**We provided a detailed description of the noncanonical transcripts within the results section, including their number (265 transcripts). We felt that the introduction is not the right place for these details.**

- Experimental design: please provide strain and sequence ID.

**The source of our OC43 virus was described in the methods section.**

- Experimental design: "following 1h adsorption". I couldn't find specific information about this, including media composition and culturing temperature (M&M section mentions culturing cells at either 37oC or 33oC).

**Thanks for noticing this. We initially neglected to include these details in the materials and methods section, but have now corrected this oversight.**

- OC43 replication and transcription kinetics: "By 6 hpi, most reads mapped to the 3' end of the viral genome". More accurately, "by 6 hpi, more reads mapped to nucleoprotein (N) sgRNA as compared to any other viral transcripts". This is consistent with results from related coronaviruses-please cite relevant literature.

**In the original text, we acknowledged that the elevated read density at the 3’ end is due to sgRNA production. The sentence in whole reads: “By 6 hpi, most reads mapped to the 3′ end of the viral genome, *consistent with the onset of sgRNA production*.” (italics added). We have now added the relevant literature.**

- OC43 replication and transcription kinetics: "The remaining reads were divided amongst 265 noncanonical sgRNAs, most of which lacked any substantial coding potential". This statement described the results of transcriptome and not translatome analysis. How could such a conclusion be drawn without the ribo-seq data?

**Coding potential is determined by the length of the ORF, and can thus be inferred from transcriptomic data. Here, the large majority of noncanonical transcripts have only very short ORFs, and thus limited coding potential. For example, for the eight most abundant noncanonical sgRNAs, the initial ORF was no longer than 16 amino acids (Fig. S3c).**

**Riboseq data addresses a different question, namely, how much is a given ORF translated? But this question is distinct from that of coding potential.**

What about cryptic translation initiation sites and other noncanonical mechanisms of translation?

**This is an interesting question, but unfortunately, we are unable to say much about translation of the noncanonical transcripts. The fundamental problem is that large portions of the noncanonical transcripts overlap in sequence with the canonical transcripts, and the short-read lengths inherent to ribosome profiling means that it’s very difficult to unambiguously map RPFs specifically to noncanonical transcripts.**

- Quality control for cellular and viral Riboseq: callouts to Fig. 3c, S5b, S5c-d are confusing. Where are the reads "excluded from the 3'UTR"?

**We reproduce Fig. S5c below, and have highlighted the 3’UTR region with a red box. At 6 hpi, this region is largely free of Riboseq reads, but this is not the case at subsequent timepoints.**

What is the "large increase in reads mapping to ORF1B"?

**We reproduce Fig. S5b below, and have highlighted ORF1B with a red box. At 6hpi, this region is largely free of Riboseq reads, but this is not the case at subsequent timepoints.**

Reviewer #3 (Significance (Required)):

This work is of high quality and can likely be used as a resource for basic research into virus-host interactions in coronavirus infection. The methodologies utilized are adequate and were previously applied to other coronaviruses. The major strength of the manuscript is the meticulous characterization of RNA dynamics, including transcription and translation, in OC43 infection. The major limitations are the density of data, lack of focus, flow of the text and incomplete validations.
